# Identification of the main venom protein components of Aphidius ervi, a parasitoid wasp of the aphid model Acyrthosiphon pisum

**DOI:** 10.1186/1471-2164-15-342

**Published:** 2014-05-06

**Authors:** Dominique Colinet, Caroline Anselme, Emeline Deleury, Donato Mancini, Julie Poulain, Carole Azéma-Dossat, Maya Belghazi, Sophie Tares, Francesco Pennacchio, Marylène Poirié, Jean-Luc Gatti

**Affiliations:** INRA, ISA, UMR 1355, Evolution et Spécificité des Interactions Multitrophiques (ESIM), Sophia Antipolis, 06903 France; Université Nice Sophia Antipolis, ISA, Sophia Antipolis, 06903 France; CNRS, ISA, UMR 7254, Sophia Antipolis, 06903 France; Dipartimento di Agraria, Laboratorio di Entomologia “E. Tremblay”, Università degli Studi di Napoli “Federico II”, Portici, Napoli, Italy; Commissariat à l’Energie Atomique (CEA), Institut de Génomique (IG), Génoscope, Evry, France; Faculté de Médecine - Secteur Nord, CNRS, Aix-Marseille Université, UMR 7286, CRN2M, Centre d’Analyses Protéomiques de Marseille (CAPM), 51, bd Dramard, Marseille, France; CNRS FRE 3498 EDYSAN, Bio-écologie des Insectes Phytophages et Entomophages (BIPE), Université de Picardie Jules Verne (UPJV), Amiens, France; Evolution and Specificity of Multitrophic Interactions (ESIM), UMR 1355 “Sophia Agrobiotech Institute” (ISA), Institut National de la Recherche Agronomique, INRA PACA, 400 route des Chappes, Sophia Antipolis, 06903 France

**Keywords:** Parasitoid wasp, Aphid, *Acyrthosiphon pisum*, *Aphidius ervi*, Venom proteins, Virulence, γ-glutamyl transpeptidase, Cystein-rich peptides

## Abstract

**Background:**

Endoparasitoid wasps are important natural enemies of the widely distributed aphid pests and are mainly used as biological control agents. However, despite the increased interest on aphid interaction networks, only sparse information is available on the factors used by parasitoids to modulate the aphid physiology. Our aim was here to identify the major protein components of the venom injected at oviposition by *Aphidius ervi* to ensure successful development in its aphid host, *Acyrthosiphon pisum*.

**Results:**

A combined large-scale transcriptomic and proteomic approach allowed us to identify 16 putative venom proteins among which three γ-glutamyl transpeptidases (γ-GTs) were by far the most abundant. Two of the γ-GTs most likely correspond to alleles of the same gene, with one of these alleles previously described as involved in host castration. The third γ-GT was only distantly related to the others and may not be functional owing to the presence of mutations in the active site. Among the other abundant proteins in the venom, several were unique to *A. ervi* such as the molecular chaperone endoplasmin possibly involved in protecting proteins during their secretion and transport in the host. Abundant transcripts encoding three secreted cystein-rich toxin-like peptides whose function remains to be explored were also identified.

**Conclusions:**

Our data further support the role of γ-GTs as key players in *A. ervi* success on aphid hosts. However, they also evidence that this wasp venom is a complex fluid that contains diverse, more or less specific, protein components. Their characterization will undoubtedly help deciphering parasitoid-aphid and parasitoid-aphid-symbiont interactions. Finally, this study also shed light on the quick evolution of venom components through processes such as duplication and convergent recruitment of virulence factors between unrelated organisms.

**Electronic supplementary material:**

The online version of this article (doi:10.1186/1471-2164-15-342) contains supplementary material, which is available to authorized users.

## Background

Aphids are Hemipteran pests responsible for major agricultural losses, notably due to vectored viral pathogens. They also have peculiar and poorly understood ecological and evolutionary features, which offer unparalleled opportunities to address evolutionary issues. More particularly, their tight association with bacterial symbionts makes them an ideal model to study the evolution of the immune system and the modulation of immune interactions [1–3]. Aphids can be attacked by various natural antagonists including endoparasitoid braconid wasps from the subfamily *Aphidiinae*. These solitary parasitic wasps lay eggs inside the body of host juvenile stages or adults. The hatching larva then develop through three larval stages to become a pupa, protected inside the hardened host body called “mummy”, from which an adult wasp will emerge [4].

*Aphidius ervi* is a widely used biological control agent that parasitizes several Macrosiphinae aphid species, including the pea aphid model *Acyrthosiphon pisum*[5, 6]. To ensure development inside the host, *A. ervi* regulates its development and metabolism and possibly evades or overcomes its immune response. Its success relies on the injection of venom at oviposition, as well as the release in the host of teratocytes, cells that derive from the dissociation of a membrane surrounding the embryo [7–10]. Until now, the physiological effects observed in the host are mainly associated with parasitoid nutrition. Venom injection, for instance, induces the degeneration of host ovaries and the arrest of its reproduction, thus redirecting host nutritional resources to the developing parasitoid larva [11–13]. In contrast, egg encapsulation has seldom been reported for aphid parasitoids and whether they may suppress or evade host immune response, as described for most parasitoids of Diptera and Lepidoptera [14], remains to be determined.

Despite the high amount of data on *A. ervi* behavior and physiology, only sparse information is yet available on its venom molecular composition. More surprisingly, there are no data on venom of other parasitoids of aphids and more generally of hemipteran hosts although they include many pests of remarkable economic importance. Until now, the only factor identified from the venom of *A. ervi* is a γ-glutamyl transpeptidase (γ-GT) that was named Ae-γ-GT [13, 15]. γ-GTs enzymes play a pivotal role in glutathione metabolism by hydrolyzing and transferring the γ-glutamyl moiety from glutathione (GSH) to various acceptors [16]. Although γ-GTs are usually membrane-bound proteins, Ae-γ-GT was found as a soluble enzyme of 57 kDa (36 and 19 kDa subunits) in venom. It was also shown to be involved in castration of its aphid host possibly because it may interfere with the delicate balance of glutathione, causing oxidative stress in ovarian cells and triggering fatal apoptosis of ovaries and early aphid embryos [13].

To identify the main *A. ervi* venom protein components, we performed a large-scale analysis using a combined transcriptomic and proteomic approach. Such broad approaches recently allowed thorough investigations of venom components in several parasitoid species, thus improving our knowledge of their nature and diversity [17–26]. The present study is the first in-depth venom analysis of a parasitoid of Hemiptera, as well as of a braconid parasitoid devoid of polydnaviruses (PDVs), key factors of host regulation in several braconid and ichneumonid species [27]. Comparison of venom data sets for *A. ervi* and PDV-associated braconid wasps, such as *Chelonus inanitus*[25], *Microctonus* sp. [19] and *Microplitis demolitor*[17], will provide insights on how the use of various parasitism strategies impacts venom evolution. Although we identified a large number of transcripts and proteins, we have focused our analysis on the major venom components since they are the most likely involved in parasitism success [18, 20].

## Results and discussion

### Identification of the main secreted proteins in A. ervi venom through a combined transcriptomic and proteomic approach

The transcriptomic analysis was performed on a French (FR) and an Italian (IT) *A. ervi* strain, using cDNA libraries from venom apparatus (glands and associated reservoirs). As our objective was to identify the major venom proteins, and since no reference genome was available, we decided to use the Sanger technology to produce long, high quality sequences (Additional file 1: Figure S1). The obtained number of sequences was approximately five times higher for the FR library than for the IT library (Additional file 2: Table S1). Tests of assembly performed on the pool of all IT and FR ESTs, using different parameters, revealed that a large part of the ESTs were shared between the IT and FR libraries. Moreover, GO terms comparison on the trimmed ESTs suggested a similar distribution for the two libraries (Additional file 3: Figure S2). The final assembly, therefore made using all pooled ESTs and default parameters, yielded a total of 1911 unisequences (unique sequences corresponding to either contigs or singletons), with a high level of redundancy (Additional file 2: Table S1). As expected from the relative number of sequences, a majority of IT ESTs (58%) were found in mixed contigs, whereas a majority of FR ESTs (61%) were found in the FR library only (Additional file 2: Table S1). Among the 42 abundant transcripts (represented by more than 10 ESTs), nearly 80% were mixed contigs suggesting a rather similar venom composition in the *A. ervi* strains (Additional file 2: Table S1). Functional annotation was performed using (i) sequence similarity searches against public databases as well as the main available predicted insect proteomes and (ii) automated open reading frame (ORF) prediction, followed by search for signal peptide and InterPro domains on the translated sequences (Additional file 1: Figure S1). As already evidenced in previous venom analyses [18, 22, 25], more than 60% of unisequences had no significant similarity in databases and could not be assigned an InterPro annotation (Additional file 2: Table S1 and Additional file 4: Table S2).

**Figure 1:**
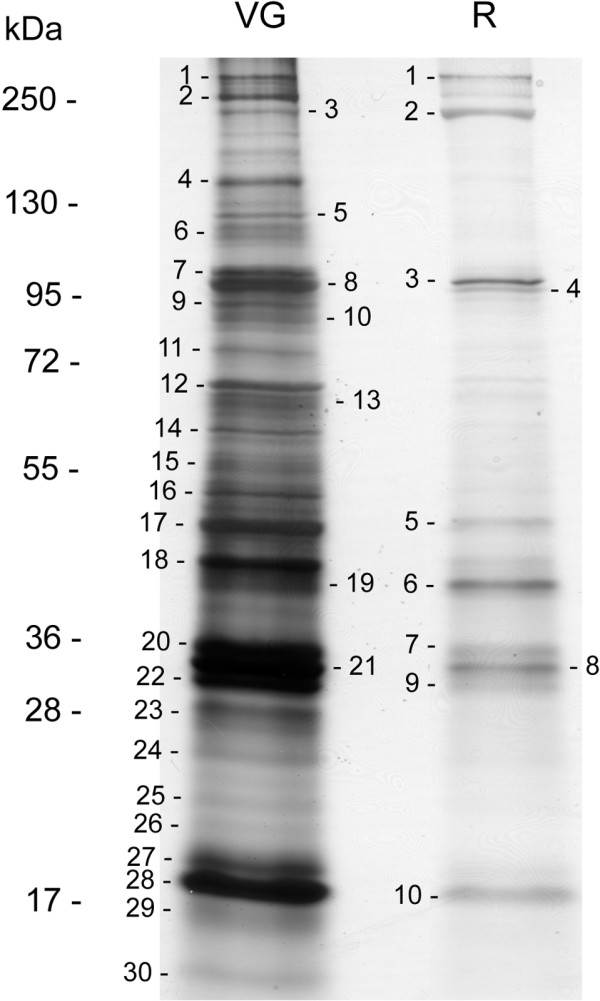
**Comparison of venom gland and reservoir protein profiles, and proteomic analysis.** Proteins from *A. ervi* venom glands and reservoirs were separated on a 6-16% SDS-PAGE under reducing conditions and visualized by silver staining. All stained protein bands numbered on the gel were excised and submitted for protein identification by LC-MS-MS. Molecular mass is in kDa.

**Figure 2:**
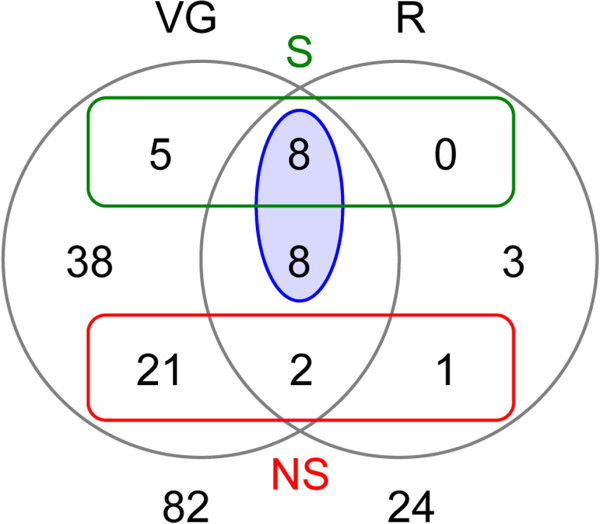
**Venn diagram showing the repartition of unisequences found in proteomics between venom glands (VG) and reservoirs (R).** The green and red rectangles highlight the number of unisequences for which sequence was complete and that were predicted to be secreted (S) or predicted not to be secreted (NS), respectively. The blue ellipse corresponds to considered “putative venom proteins”.

The proteomic analysis was performed on the *A. ervi* FR strain, on venom gland and reservoir samples separately. On a 6-16% SDS-PAGE, the protein content of each compartment was resolved in bands from less than 15 kDa to more than 250 kDa (apparent molecular mass, Figure 1). As expected, most of the major bands observed in the venom glands were also detected in the reservoirs, despite an overall quantitative difference in protein load between these tissues due to the small amount of venom in the reservoirs. All the major bands on electrophoretic patterns as well as a number of minor bands (a total of 30 bands for the venom glands, 10 for the venom reservoirs) were excised, and tryptic peptides were analyzed by LC-MS-MS (Additional file 5: Table S3). The integrated analysis of transcriptomic and proteomic data resulted in 86 matches, among which a putative function was found for 65 unisequences (Table 1 and Additional file 6: Table S4). However, most of the unisequences found in proteomics were detected in the venom gland only (Figure 2), and many of them probably corresponded to cellular proteins (e.g. ribosomal proteins) (Table 1 and Additional file 6: Table S4). Some of the cellular proteins also found in the reservoir had a predicted muscular function (e.g. actin, with as much as 30 peptide matches, paramyosin and spectrin; Additional file 6: Table S4), thus supporting the main role of the reservoir in pumping and injecting venom during oviposition. The presence of cellular proteins likely resulted from tissue contamination since cell leakage was difficult to prevent during venom collection from the gland (two filaments with a thin canal; Additional file 1: Figure S1), while venom could only be extracted from the reservoir by crushing the tissues. Although this likely resulted in under-evaluating their number, we therefore only considered as putative venom proteins the 16 unisequences (i) found in proteomics in both venom glands and reservoirs and (ii) predicted to be secreted or for which secretion could not be predicted due to the incompleteness of the sequence (Figure 2 and Table 2).

**Table 1:** **Classification of unisequences found in proteomics according to putative function. Putative venom proteins are highlighted in italic**

Putative function	Unisequences^a^				EST^b^			Mascot^c^		Signal peptide^d^
	Total	FR + IT	FR	IT	Total	FR	IT	VG	R	
14-3-3 family	1		1		7	7		1		No
Actin	2			2	2		2	3	30	No
Calpain	2		2		3	3		2		?
Elongation factor 1-alpha	1		1		4	4		1		?
*Elongation factor 2*	1		1		6	6		3	1	?
*Endoplasmin*	3	2	1		19	16	3	60	8	Yes^#^
Fatty acid synthase	1		1		1	1		1		?
*γ-glutamyl transpeptidase*	3	3			539	463	76	177	25	Yes
Glycoside hydrolase domain-containing protein	2		2		15	7		2		?
Heat shock protein 70	6	2	4		25	22	3	59		Yes^#^
Hypoxia up-regulated protein 1	1		1		2	2		1		?
Inositol-3-phosphate synthase	2		2		2	2		2		?
*Leucine rich repeat domain-containing protein*	2	1	1		30	28	2	12	4	Yes^#^
Low-density lipoprotein receptor-related protein 2	1	1			13	12	1	1		?
Neprilysin-like	1		1		2	2		1	2	?
Paramyosin, long form-like	1			1	1		1		1	?
Peptidyl-prolyl cis-trans isomerase	1		1		3	3		4		?
Protein disulfide isomerase	2		2		2	2		8		?
Rab GTPase family	1		1		2	2		2		?
Ribosomal protein	19	3	14	2	42	36	6	30		?
*Serine protease homologue*	5	3	2		97	78	19	22	1	Yes^#^
*Serpin*	2		2		26	26		10	3	Yes^#^
Spectrin	1		1		1	1			1	?
Staphylococcal nuclease domain-containing protein	1		1		1	1		2		?
Transcription factor BTF3-like	1		1		1	1		1		?
V-type proton ATPase catalytic subunit A	1		1		1	1		1		No
Vesicular integral-membrane protein VIP36-like	1		1		1	1		1		?

^a^Uniseq: number of unisequences.

^b^EST: number of ESTs.

^c^Mascot: number of matches with peptides.

^d^SP: Prediction of peptide signal by TargetP. A ? means that prediction of secretion could not be performed due to the incompleteness of the sequence(s). A # indicates that a SP was predicted for some but not all unisequences.

**Table 2:** **Putative venom proteins classified according to the number of ESTs**

Sequence name	Putative function	EST^a^			Mascot^b^		Signal peptide^c^
		Total	FR	IT	VG	R	
CL1Contig7	γ-glutamyl transpeptidase	319	317	2	133	16	Yes
CL1Contig2	γ-glutamyl transpeptidase	120	50	70	33	8	Yes
CL1Contig6	γ-glutamyl transpeptidase	100	96	4	11	1	Yes
CL9Contig1	Serine protease homologue	41	34	7	2	1	Yes
CL2Contig1		30	24	6	18	1	Yes
CL18Contig1	Serpin	25	25		9	2	Yes
CL3Contig3	Leucine rich repeat domain-containing protein	23	21	2	7	3	Yes
CL28Contig1	Endoplasmin	15	13	2	52	6	Yes
CL2Contig11		14	14		18	1	?
CL3Contig5	Leucine rich repeat domain-containing protein	7	7		5	1	?
CL56Contig1	Elongation factor 2	6	6		3	1	?
CL257Contig1	Neprilysin-like	2	2		1	2	?
CL209Contig1	Endoplasmin	2	2		6	2	?
CL296Contig1		2	2		3	1	?
aar0aka7ya15cm1.1		1	1		7	1	?
aar0aka8ya02cm1.1	Serpin	1	1		1	1	?

^a^EST: number of ESTs.

^b^Mascot: number of matches with peptides.

^c^SP: Prediction of peptide signal by TargetP. A ? means that prediction of secretion could not be performed due to the incompleteness of the sequence.

### Putative function of the main identified A. ervi venom proteins

A putative function was predicted for 12 of the 16 unisequences considered as venom proteins (Table 2 and Additional file 6: Table S4). Among them, 7 sequences were considered as abundant based on the number of ESTs. Moreover, we generally observed a good correlation between the number of ESTs and the number of matches with mass spectrometry peptides, although the proteomic analysis was not strictly quantitative. The abundant unisequences were (i) 3 γ-GTs, (ii) 1 serine protease homologue, (iii) 1 leucine rich repeat domain-containing protein, (iv) 1 serpin and (v) 1 endoplasmin (Table 2 and Additional file 6: Table S4). Real-time PCR analysis of the relative expression of a selection of these unisequences (1 γ-GT, the serine protease homologue, the serpin) evidenced a venom tissue-specific expression (Table 3 and Additional file 7: Figure S3), as expected for putative venom proteins.

**Table 3:** **Mean relative expression in venom apparatus and bodies without venom apparatus for a selection of unisequences coding for putative venom proteins and toxin-like peptides**

Unisequence name	Putative function	Number of ESTs	Venom apparatus (SE)	Bodies w/o venom apparatus (SE)
CL1Contig6	γ-glutamyl transpeptidase	100	811.62 (107.47)	2.51 (1.42)
CL9Contig1	Serine protease homologue	41	562.35 (6.93)	1.87 (0.9)
CL18Contig1	Serpin	25	637.9 (86.78)	2.77 (1.75)
CL1Contig4	Toxin-like	60	451.74 (39.01)	1.91 (1.07)
CL1Contig1	Toxin-like	33	439.92 (17.43)	2.17 (1.22)
CL1Contig5	Toxin-like	9	1057.45 (132.07)	1.87 (0.9)

#### γ-glutamyl transpeptidases

Our analysis led to identification of three different γ-GTs in *A. ervi* venom (Additional file 8: Figure S4), including Ae-γ-GT, which represent by far the most abundant proteins (Table 2 and Additional file 6: Table S4). γ-GTs are found in bacteria, plants, and animals. They are key-enzymes in glutathione (GSH) homeostasis that catalyze the transfer of the γ-glutamyl moiety from GSH, as well as other γ-glutamyl compounds, to amino acids or GSH itself [16]. γ-GTs thus play an important role in intracellular redox status, cytosolic iron metabolism, and inflammation. Although considered as heterodimeric cell-surface enzymes, γ-GTs are also found under soluble circulating forms in body fluids, as Ae-γ-GT in *A. ervi* venom [13]. Accordingly, all three *A. ervi* venom γ-GTs identified in our analysis were predicted to contain a peptide signal and thus could be secreted or shed from the cell surface (Table 2 and Additional file 6: Table S4).

**Figure 3:**
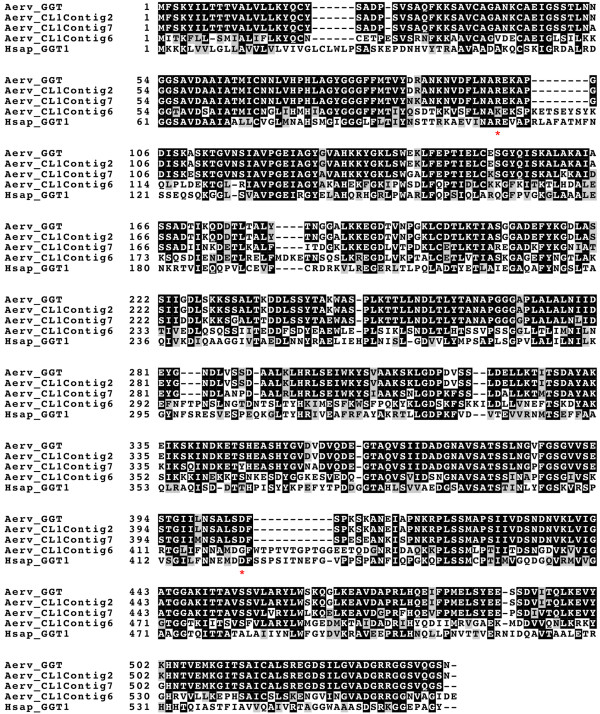
**Multiple alignment of γ-GT sequences.** The three *A. ervi* γ-GT sequences identified were aligned with the published *A. ervi* γ-GT sequence [GenBank: CAL69624] and the human γ-GT1 sequence [Swiss-Prot:P19440]. Residues identical or similar are highlighted in black and grey, respectively. Stars indicate mutations in the Aerv_CL1Contig6 that were described to affect the enzymatic activity of human γ-GT1. Aerv, *A.ervi*; Hsap, *H. sapiens.*

**Figure 4:**
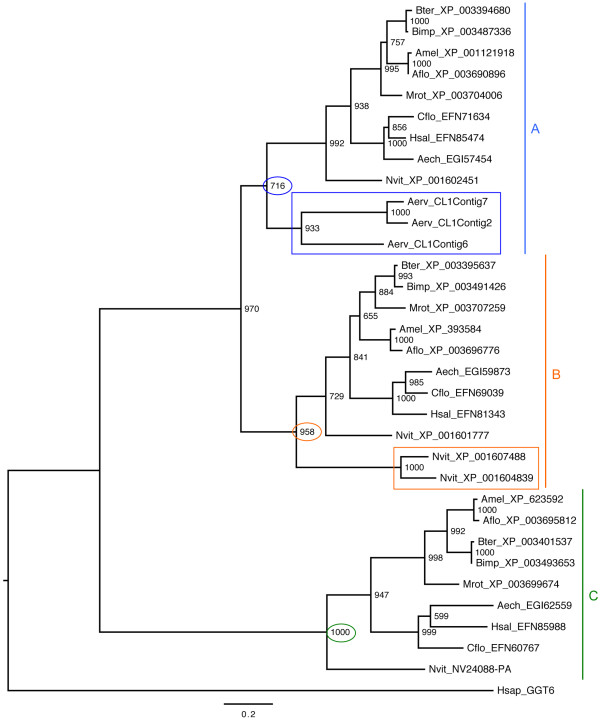
**Maximum-likelihood phylogenetic tree of hymenopteran γ-GT sequences.** The blue, orange, and green vertical lines correspond to the three major clades (A, B and C) obtained for hymenopteran γ-GT sequences. *A. ervi* and *N. vitripennis* venomous γ-GT sequences are marked with blue and orange rectangles respectively. Numbers at corresponding nodes are bootstrap support values (1000 bootstrap replicates). The outgroup is the human γ-GT6 sequence [Swiss-Prot: Q6P531]. Aech, *Acromyrmex echinatior*; Aerv, *Aphidius ervi*; Aflo, *Apis florea*; Amel, *Apis mellifera*; Bimp, *Bombus impatiens*; Bter, *Bombus terrestris*; Cflo, *Camponotus floridanus*; Hsal, *Harpegnathos saltator*; Hsap, *Homo sapiens*; Mrot, *Megachile rotundata*; Nvit, *Nasonia vitripennis.*

Among the three *A. ervi* γ-GTs, the amino acid sequences of CL1Contig2 and CL1Contig7 were respectively identical and very close (87% identity) to the Ae-γ-GT previously published sequence (Figure 3). Interestingly, CL1Contig2 was the most abundant venom γ-GT in the Italian strain, while CL1Contig7 was the most abundant in the French strain (Table 2 and Additional file 6: Table S4) suggesting they might be alleles occurring at different frequencies. This is in agreement with our observation of a rapid decrease in the frequency of CL1Contig2 in the French strain under our rearing conditions (data not shown). Whether these two γ-GTs similarly contribute to induce apoptosis in the pea aphid ovarian cells remains to be investigated. Strikingly, CL1Contig6, which is highly expressed in the French strain, shares only 51% identity to the published sequence (Figure 3). Moreover, although overexpressed in the venom apparatus (Table 3 and Additional file 7: Figure S3), it contains two mutations previously described to strongly reduce the enzymatic activity of human γ-GT1 [28, 29]. The corresponding residues are otherwise conserved in other hymenopteran GGT sequences belonging to the same clade (Figures 3, 4, and Additional file 9: Figure S5). This raises the questions whether it is a fully active γ-GT and which role it may play in *A. ervi* parasitism success.

Using Hymenopteran databases, we were able to identify three distinct types of non-venomous γ-GTs forming three distinct phylogenetic clades (Figure 4). Interestingly, the three *A. ervi* venomous γ-GTs group with clade A, while the two γ-GTs recently identified in *N. vitripennis* venom, which function is yet unknown [22], group with clade B (Figure 4). The venomous γ-GTs of these parasitoid species may thus originate from distinct duplication events of two different genes coding for γ-GT "classical" proteins.

Interestingly, γ-GTs can be used by bacteria as virulence determinants. For instance, a γ-GT contributes to *Helicobacter pylori* tolerizing effects on murine dendritic cells and suppressive activity on T cells, mainly via the depletion of glutamine [30]. *Campylobacter jejuni* virulence and colonization of the avian gut is also dependent upon the activity of a γ-GT that participates in its cell apoptosis-inducing activity by a yet unknown mechanism [31]. Whether venom γ-GTs can also act as virulence factors in parasitoids remains to be assessed.

#### Serine protease homologues

Serine proteases are endopeptidases whose active site contains a serine and which are involved in various biological processes, including immunity. Serine protease homologues (SPHs) lack one or more residues essential for catalytic activity [32] and do not have proteolytic activity. The five unisequences identified in *A. ervi* venom apparatus libraries, with a total of 97 ESTs (Table 2 and Additional file 6: Table S4), encode different SPHs (11% to 90% sequence identity), all with mutation(s) on the catalytic triad (Additional file 10: Figure S6). The four SPHs for which the 5' coding sequence was complete contain a signal peptide, suggesting that they are secreted. Based on our criteria, only one of these SPHs could be classified as a venom protein: it was considered as abundant, with a total of 41 ESTs, and it was found in proteomics in the venom reservoir (Table 2 and Additional file 6: Table S4). Besides, it was specifically overexpressed in the venom apparatus (Table 3 and Additional file 7: Figure S3). However, two other SPHs were also abundant although not found in the reservoir. Members of the serine protease family have been described in the venom of several other parasitoids [22, 25, 26, 33, 34], the most studied being Vn50, secreted in *Cotesia rubecula* venom and devoid of serine protease activity. Vn50 acts as an inhibitor of the hemolymph melanization in the host *Pieris rapae*, presumably by competing with host serine protease homologs for binding to proPO, while remaining non-cleaved and stable in the haemolymph [33, 35].

#### Leucine-rich repeat domain-containing proteins

Two different unisequences encoding leucine-rich repeat (LRR) domain-containing proteins, never described yet in a parasitoid venom, were found in our analysis with a total of 30 ESTs (Table 2 and Additional file 6: Table S4). Interestingly, the sequences were mostly found in the French library since only 2 ESTs came from the Italian strain (Table 2 and Additional file 6: Table S4). The unisequence that was complete contains a signal peptide at N-terminus suggesting the secretion of the protein. It also contains a total of 8 canonical LRR motifs separated by one to three amino acids (Additional file 11: Figure S7), although a manual analysis suggested the presence of six to seven additional, though cryptic, repeats. Interestingly, the conserved LxxLxLxxNxLxxLxxxxF sequence present in the 8 canonical LRR motifs is similar to the one described in Toll Like Receptors (TLRs) [36]. However, *A. ervi* predicted proteins only contain the LRR domain by contrast to the majority of TLRs that are multidomain proteins. With the loss of all but the LRR domain, *A. ervi* venom proteins might act as scavengers for the pea aphid TLRs, thus impairing the host immune response via the Toll pathway. Interestingly, the use of a truncated single-domain protein as a virulence factor has already been described for a parasitoid venom protein [37].

#### Serpins

Serpins (serine protease inhibitors) are a large family of functionally diverse protease inhibitors. They share a conserved structural architecture with an exposed reactive center loop (RCL) of about 20 amino acids, which acts as bait for target serine proteases [38]. Interestingly, the involvement of a *Leptopilina boulardi* venom serpin in suppressing host immunity was already demonstrated. LbSPNy indeed prevents melanization in the *Drosophila* host through inhibition of PO activation [39]. More recently, serpins were described in the venom of *Hyposoter didymator*[20] and *M. demolitor*[17] but their role in parasitism success remains unknown. The two identified *A. ervi* serpin-like unisequences were both found in the French library only and detected in the venom reservoir (Table 2 and Additional file 6: Table S4). However, only one of these unisequences, overexpressed in the venom apparatus (Table 2 and Additional file 7: Figure S3) could be considered as abundant with 25 ESTs. Interestingly, both serpins lack the consensus hinge sequence (Additional file 12: Figure S8) essential for the conformational change involving the RCL and necessary to inhibit the target protease [38]. The identified venom protein thus probably belongs to the group of non-inhibitory serpins that have varied roles such as chaperones or transport molecules [40].

#### Endoplasmin

Endoplasmin, which belongs to the heat shock protein 90 family, is a molecular chaperone located in the endoplasmic reticulum (ER) and involved in the final processing and export of secreted proteins [41]. Although three incomplete endoplasmin-like unisequences were identified in *A. ervi*, they match to different regions of the same *N. vitripennis* endoplasmin sequence (Additional file 13: Figure S9) and thus likely correspond to a single gene. The endoplasmin protein was considered as abundant based on the number of ESTs and accordingly detected at high levels in *A. ervi* venom reservoir (Table 2 and Additional file 6: Table S4). The *A. ervi* sequence contains the C-terminal HEEL motif that normally prevents secretion of ER-resident proteins (Additional file 13: Figure S9). However, this ER retention is not absolute [42]. Endoplasmin has never been described yet in any parasitoid venom but it has been associated with the secretion of pancreatic lipases and their further internalization by intestinal cells [43]. This suggests a possible role of this chaperone in the secretion, stabilization, transport and host cell targeting of the different *A. ervi* venom proteins.

Two other unisequences having putative functions in venom were found in low abundance in *A. ervi* based on the number of ESTs: (i) 1 elongation factor and (ii) 1 neprilysin-like protein (Table 2 and Additional file 6: Table S4).

#### Elongation factor

One transcript of elongation factor 2 (EF-2), an essential protein that regulates the process of polypeptide elongation during translation, was found in low abundance in the *A. ervi* French library (Table 2 and Additional file 6: Table S4). Although EF-2 was also found in the reservoir (1 peptide match), the sequence was not complete and accurate prediction of its secretion could not be performed. To our knowledge, there is no report yet of EF-2 involvement either as a virulence factor or a venom protein. Interestingly, elongation factor 1-alpha (EF-1α) was found in the venom of another parasitoid, *L. heterotoma*[18], but its role in the host-parasitoid interaction is also unknown. EF-1α was identified as a secreted candidate virulence factor in *Leishmania* protozoan parasites, being possibly involved in the induction of macrophage deactivation through direct binding and activation of a specific host tyrosine phosphatase [44].

#### Neprilysin-like (NEP-like)

One unisequence encoding a neprilysin-like protein was found in low abundance (2 ESTs and 2 peptide matches in the venom reservoir) in the *A. ervi* French library (Table 2 and Additional file 6: Table S4). NEP-like proteins are zinc-dependent metalloproteases (ectopeptidases) belonging to the M13 peptidase family. They are involved in the degradation of a number of regulatory peptides in the nervous or immune system of mammals [45] and insects [46]. Although typically membrane-bound, ectopeptidases such as NEP may also be shed from the membrane through a proteolytic process and found in the surrounding fluid [47]. NEP-like proteins were detected in the venom of the parasitoids *L. boulardi*[18], *Microctonus hyperodae*[19], *H. didymator*[20] and *M. demolitor*[17], and were also found associated with the VLPs produced in the ovary of *V. canescens*[48]. Although the role of soluble ectopeptidases is still not understood, NEP-like proteins have been hypothesized to modulate the host immune system by degrading immune-specific peptides [48].

### Expression of genes encoding cystein-rich toxin-like peptides in A. ervi venom gland

Three unisequences coding for small cysteine-rich peptides predicted to be secreted were found in our transcriptomic analysis and demonstrated to be specifically expressed in the venom apparatus (Table 3 and Additional file 7: Figure S3). Although two of these, CL1Contig4 (60 ESTs) and CL1Contig1 (33 ESTs) were considered as abundant, the small molecular weight of the predicted mature peptides (from 2.83 to 3.88 kDa; Table 4) precluded their analysis by SDS-PAGE proteomics (Additional file 6: Table S4).

**Table 4:** **Summary of the toxin-like peptides analysis and comparison with defensin-NV (Ye et al.**[51]**)**

	CL1Contig4	CL1Contig1	CL1Contig5	Defensin-NV
**Number of ESTs**				
**Total**	60	33	9	
FR	37	27	6	
IT	23	6	3	
Signal peptide				
TargetP	Yes	Yes	Yes	
Reliability	Strongest	Strongest	Strongest	
Sequence length				
Complete (aa)	60	60	51	
Mature (aa)	36	36	27	
Molecular weight				
Mature (kDa)	3.88	3.79	2.83	
Similarity searches				
Swiss prot best hit	U8-theraphotoxin-Cj1a	Conotoxin Vi11.3	Conotoxin AbVIN	Defensin-1
Accession	B1P1C0	C7DQX8	Q9TVQ6	Q5J8R1.1
Organism	*Chilobrachys jingzhao*	*Conus vitulinus*	*Conus abbreviatus*	*Apis mellifera carnica*
Molecular function	Toxin	Toxin	Toxin	Defensin
Domain	Knottin	Knottin	Knottin	-
E-value	0.026	2.9	0.34	2e-21
Toxin prediction				
ClanTox	Toxin-like	Toxin-like	Toxin-like	Toxin-like
Reliability	Strongest	Strongest	Strongest	Strongest
Knottin prediction				
Knoter1D	Ambiguous knottin	Ambiguous knottin	Putative knottin	Not a knottin
AMP Prediction				
AMPer	Antimicrobial peptide Alo-3	Beta-defensin	Beta-defensin	Defensin
Lowest HMM E-value	0.0016	0.005	0.0054	2e-20
ClassAMP	Antibacterial	Antibacterial	Antifungal	Antibacterial
Probability	0.512	0.556	0.366	0.806

**Figure 5:**
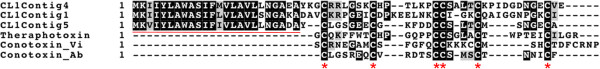
**Multiple alignment of toxin-like sequences.** The three *A. ervi* toxin-like sequences were aligned with the mature peptide sequence corresponding to each BLAST best hit (SwissProt database). Residues identical or similar are highlighted in black and grey, respectively. The predicted signal peptide is underlined in red, the six conserved cysteine residues are identified by red stars. Theraphotoxin: U8-theraphotoxin-Cj1a from *Chilobrachys jingzhao* [Swiss-Prot: B1P1C0]; Conotoxin_Vi: Conotoxin Vi11.3 from *Conus vitelinus* [Swiss-Prot: C7DQX8]; Conotoxin_Ab: Conotoxin AbVIN from *Conus abbreviatus* [Swiss-Prot: Q9TVQ6].

BLAST hits were obtained with several small animal toxins for the three unisequences, although E-values were not highly significant due to the size of the peptide sequences (Table 4). The toxin-like function of these unisequences was further confirmed using ClanTox (Table 4). Interestingly, multiple alignment revealed a highly conserved signal peptide sequence, suggesting a common evolutionary origin for the three peptides (Figure 5). By contrast, the sequences of the predicted mature peptides were strongly divergent, except for the conservation of six cysteine residues that may form three stabilizing disulfide bridges. These cysteine residues are conserved in each of the best BLAST hit for the three sequences (Figure 5), the corresponding peptides being classified as knottins, extremely stable small disulfide-rich proteins with a knotted topology [49]. Remarkably, the three *A. ervi* toxin-like peptides also possibly correspond to knottins, never described in any parasitoid venom to date, although the prediction was not fully supported (Table 4).

Few peptides have been characterized to date from parasitoid venoms. One of them is Vn1.5, a short peptide of 14 amino acids required for the expression of *Cotesia rubecula* polydnaviruses in *Pieris rapae* host hemocytes and the following inactivation of these hemocytes [50]. The *Nasonia vitripennis* venom analysis predicted occurrence of several cysteine-rich peptides with a protease inhibitor motif [22]. However, functional data on these peptides are lacking and only one defensin-like antimicrobial peptide, defensin-NV, was purified from *N. vitripennis* venom [51]. Defensin-NV is a 52 amino acid peptide with six cysteines forming three disulfide bridges that has strong antimicrobial activity against wide spectrum microorganisms, but which is not predicted as a knottin (Table 4). Another *N. vitripennis* defensin-like peptide, nasinin-3, with a similar structure but no antimicrobial activity was recently demonstrated as a potential inhibitor of host hemocytes’ melanization *in vitro.* It is however unclear whether it is found in venom [52]. Interestingly, the three *A. ervi* toxin-like peptides also share weak similarities with defensin-like antimicrobial peptides (Table 4), but their possible role as an antimicrobial factor or an inhibitor of melanization remains to be assessed.

## Conclusions

This paper reports the first identification of the main putative venom proteins of a parasitoid of aphids, *A. ervi*, using the same combined large-scale transcriptomic and proteomic approach we successfully used previously [18]. The analysis focused on a restricted number of proteins based on their predicted abundance and the occurrence of proteomic matches both in venom gland and reservoir. A total of 16 putative venom proteins were considered, a low number compared to other analyses [18–26], suggesting possible occurrence of additional low-abundant venom proteins in *A. ervi*. However, this conservative approach largely precluded misidentification of cellular proteins as venom factors. Interestingly, 12 out of the 16 considered proteins could be assigned a predicted function, in contrast to the majority of putative venom proteins in large broad analyses that did not display similarity to any known protein [18–26]. The combined analysis of two datasets corresponding to different *A. ervi* strains (French and Italian) confirmed that the major venom proteins are shared by different parasitoid populations. However, it also identified striking differences in the abundance of transcripts for some of the main unisequences such as the γ-GTs, suggesting variations in allele frequency and/or gene expression level among populations that remain to be explored.

Our study confirmed the identification of Ae-γ-GT as the most abundant protein by far in *A. ervi* venom, thus supporting its role as a key player in parasitism success [13]. In addition to an allelic form of Ae-γ-GT, we identified a divergent, possibly non-functional, γ-GT, whose biological function, if any, remains to be explored. Interestingly, we recently identified a multigenic family for a venom protein of a parasitoid of *Drosophila*, with all members except one mutated in one or more essential amino acids [18]. γ-GTs have also been observed in the venom of the ectoparasitoid *N. vitripennis,* although no information is available regarding their abundance and function. Our data nevertheless add γ-GTs as a new example of independent convergent recruitment of venom proteins in evolutionary distant parasitoid species.

Among the abundant putative venom proteins, serpins and SPHs were described in venom of other braconids and more distant parasitoid wasps, further suggesting occurrence of a conserved subset of venom proteins across parasitoid species [21, 25, 53].

Other putative venom proteins were unique to *A. ervi*, including endoplasmin or LRR domain-containing proteins, suggesting a rapid evolution of some venom components. Finally, occurrence of toxin-like cystein-rich peptides was predicted in some parasitoid species but the diversity of their nature and function remains to be explored.

One main challenge will be now to decipher the biological function of the identified venom proteins and their role in the parasitism success of *A. ervi*. This might be performed using the RNAi technique, as RNAi-mediated complete extinction of a venom protein was recently evidenced in an endoparasitoid wasp [54]. Results will open the way to a better understanding on aphid-parasitoid immune and nutritional parasitoid interactions.

## Methods

### Insect rearing

The French (FR) *A. ervi* strain was produced by mass-rearing the progeny of individuals emerged from aphid mummies provided by Biobest (Ervi-M-sytem, Orange, France). Parasitoids have been since maintained in cages on the aphid *A. pisum* LL01 clone raised on fava bean, under a 16:8 h light/dark cycle, at 18°C. The LL01 clone hosts *Buchnera aphidicola* but it is devoid of secondary symbionts. The Italian (IT) strain of *A.ervi* was reared on *A. pisum* maintained on potted fava bean plants, under the same environmental conditions as described above. Both host and parasitoid colonies were started within insects collected in Southern Italy (Eboli, SA), which were periodically bred over the years with field material originating from the same area. No ethical approval is needed for experimental work on insects such as the wasp *Aphidius ervi.*

### Collection of venom apparatus, total RNA isolation and cDNA libraries construction

The transcriptomic analysis was performed on *A. ervi* venom apparatus, corresponding to venom glands and their associated reservoirs (Additional file 1: Figure S1). Venom apparatus were dissected in Ringer’s saline (KCl 182 mM; NaCl 46 mM; CaCl_2_ 3 mM; Tris-HCl 10 mM) and stored at –80°C. Total RNA was extracted for each strain from 100 venom apparatus, using TRIzol Reagent (Invitrogen) according to manufacturer’s instructions. cDNA libraries were constructed from 1 μg of total RNA using the Creator SMART cDNA Library Construction Kit (Clontech). Ligation products were transformed into ElectroMax DH10 B *Escherichia coli* competent cells (Invitrogen).

### Sequencing, EST processing and assembly

A general overview of the sequence data processing is given in Additional file 1: Figure S1. Sanger sequencing was done on an ABI sequencer using the standard M13 forward primer and BigDye terminator cycle sequencing kit (Applied Biosystems, Foster City, CA, USA). Following a primary step of analysis of 384 clones to check the quality of each library and confirm the presence of venom protein-related sequences, a total of 6,000 clones were sequenced, 5,000 for *A. ervi* FR and 1,000 for *A. ervi* IT. FR ESTs were processed using SURF analysis pipeline tools (SURF: SeqUence Repository and Feature detection) as previously described [18]. IT ESTs were trimmed using TIGR SeqClean software. High quality trimmed ESTs longer than 100 bp from FT and IT libraries were then pooled and assembled into contigs using the TIGR-TGICL tool with different parameters. Based on the test results, the final assembly was the one performed with default parameters [55].

### Sequence annotation and analysis

To identify similarities with known proteins, the sequences of contigs and singletons were compared using the blastx algorithm against local non-redundant NR (NCBI, 2012-10-25), UniProtKB/Swiss-Prot (SIB, 2012-10-21), and insect predicted protein/proteome databases (*Acromyrmex echinatior* v3.8, *Acyrthosiphon pisum* v2.1, *Aedes aegypti* v1.3, *Anopheles gambiae* v3.6, *Apis mellifera* v4.5, *Bombyx mori*, *Drosophila melanogaster* v5.46, *Drosophila pseudoobscura* v2.28, *Nasonia vitripennis* v1.2 and *Tribolium castaneum* v20051011) with a cut-off E-value of 1e-7. ORF prediction and translation were performed using FrameDP software [56] (available at http://iant.toulouse.inra.fr/FrameDP/). Signal peptide prediction was obtained using TargetP (available at http://www.cbs.dtu.dk/services/). Search for protein domains was performed using InterProScan. Gene functions and GO terms were automatically assigned to the predicted proteins based on the identification of InterPro domains with InterProScan. Only the root domain of the hierarchical domain organization available from EBI was conserved. Comparison of GO terms between FR and IT contigs and homogenization of the annotation level were performed using the GO slim.

Multiple amino acid sequence alignments were performed using MUSCLE [57]. For phylogeny, search for Hymenopteran γ-GT sequences was performed using BLASTP at NCBI (http://www.ncbi.nlm.nih.gov/blast/). Identification of *N. vitripennis* NV24088-PA sequence was performed using HMMsearch from the HMMER package [58] with the G_glu_transpept (PF01019) HMM profile on *N. vitripennis* v1.2 proteome database. Phylogenetic analysis of γ-GT amino acid sequences was performed using maximum likelihood (ML) with PhyML [59]. ProtTest [60] was used to select the best-fit model of amino acid substitution for ML phylogeny. Leucine-rich repeats (LRRs) were predicted using ScanProsite (http://au.expasy.org/prosite/). Toxin prediction was performed using ClanTox available at http://www.clantox.cs.huji.ac.il/[61]. Knottin prediction was performed using Knoter1D available at the KNOTTIN database (http://knottin.cbs.cnrs.fr).

### SDS-polyacrylamide gel electrophoresis of venom and protein identification

The proteomic analysis was performed independently on wasp venom glands and reservoirs (Additional file 1: Figure S1). Forty *A. ervi* female venom apparatus were dissected and reservoirs were separated from the glands. Glands and reservoirs were then independently collected in 25 μl of Ringer’s solution supplemented with a protease inhibitor cocktail (Sigma). Reservoirs were solubilized immediately by mixing with 4× Laemmli buffer containing ß-mercaptoethanol (v/v), while glands were squeezed to release the venom content. The glands suspension was then centrifuged for 2 min at 500 g to remove the residual tissues, and the supernatant was carefully collected and mixed with 4× Laemmli buffer containing β-mercaptoethanol (v/v). Both reservoir and gland samples were boiled for 5 minutes. Proteins were then separated on a 6-16% linear gradient SDS-PAGE and the gel was silver stained [62]. Identification of proteins by mass spectrometry was performed on bands excised from the gel, cut into small blocks, and rinsed with water and acetonitrile prior to reduction and alkylation. Sample trypsinization was then carried out overnight at 37°C with 12.5 ng/μl trypsin (sequencing grade, Sigma). The generated peptides were sequenced by nano-LC-tandem mass spectrometry (MS/MS) (Q-TOF Ultima with a nano-electrospray ionization source, Waters/Micromass, UK) in data-dependent acquisition (DDA) mode using the five most intense parent ions. The peptides were loaded on a C18 column (XBridge™ BEH130 3,5 μm, 75 μm × 150 mm, Waters) and eluted with a 5 to 60% linear gradient at a flow rate of 200 nl/min over 90 min (buffer A: water/acetonitrile (98:2, v/v) and 0.1% formic acid; buffer B: water/acetonitrile (20:80, v/v) and 0.1% formic acid). MS/MS data analysis was performed with the Mascot software (http://www.matrixscience.com) licensed in house, using the contig sequences of the Aphidius mixed library and non-redundant NR (NCBI). Peak lists generated for individual bands from the same gel lane were merged together into a single file before databank search submission. Data validation criteria were (i) one peptide with individual ion score above 50 (the mascot significant identity threshold corresponding to p < 0.005 is 38 in our case) or (ii) at least two peptides of individual ion score above 20 (corresponding to 1% probability that a peptide spectrum match is a random event). The mascot score was calculated as -10Log(P). The calculated FDR (based on an automatic decoy database search) was lower than 1%: FDR = 0.23% and 0% for venom glands and reservoirs respectively.

### Quantitative real-time RT-PCR

Total RNA was isolated either from dissected venom apparatus or from the rest of the female bodies (without venom-producing tissues) using the TRIzol reagent (Invitrogen), and reverse-transcribed using the iScript cDNA Synthesis Kit (BioRad). qPCR reactions were then carried out on an Opticon monitor 2 (BioRad) using the Absolute qPCR SYBR MasterMix Plus for SYBR Green I No ROX (Eurogentec). Primer pairs are listed in Additional file 14: Table S5. PCR conditions were as follows: 50°C for 2 min, 95°C for 10 min, and 40 cycles of 95°C for 30 s, 60°C for 30 s and 68°C for 30 s. Each reaction was performed in triplicate and the mean of three independent biological replicates was calculated. All data were normalized using RPL19 and RPL23 as controls and results were analyzed using Qbase Software (Ghent University, Ghent, Belgium).

## Availability of supporting data

All trimmed ESTs for *A. ervi* FR and *A. ervi* IT are available in the NCBI dbEST repository with the following accession numbers: JZ569599 - JZ573851. The assembled transcripts corresponding to putative venom proteins have been deposited in GenBank under Transcriptome Shotgun Assembly accession number GBCU00000000. The version described in this paper is the first version, GBCU01000000.

## Electronic supplementary material

Additional file 1: Figure S1: Schematic representation of the combined large-scale transcriptomic and proteomic approach. Upper picture; venom apparatus of *A. ervi.* VG: venom gland; R: reservoir; DG: Dufour gland. (TIFF 982 KB)

Additional file 2: Table S1: General features of the *A. ervi* cDNA FR and IT libraries, results of assembly of pooled FR and IT sequences and similarity searches. (DOC 60 KB)

Additional file 3: Figure S2: Interlibrary comparison of the representation of GO categories. Distribution of the number of unisequences associated with GO terms for the FR and IT libraries. The difference in the number of FR and IT sequences was taken into account using the ratio of the number of trimmed sequences between FR and IT. (TIFF 838 KB)

Additional file 4: Table S2: Most abundantly represented transcripts (>10 ESTs) in the *A. ervi* cDNA libraries. Mixed contigs are highlighted in gray. (XLS 41 KB)

Additional file 5: Table S3: Matrix scores and peptides identified by mass spectrometry (R). (XLS 256 KB)

Additional file 6: Table S4: Unisequences found in proteomics. Mixed contigs are highlighted in gray. (XLS 52 KB)

Additional file 7: Figure S3: Mean relative expression in venom apparatus and bodies without venom apparatus. qRT-PCR experiments were performed for a selection of unisequences coding for putative venom proteins and toxin-like peptides. All data were normalized using RPL19 and RPL23 controls. (TIFF 526 KB)

Additional file 8: Figure S4: Specific peptides identified in proteomics for the three *A. ervi* venom γ-GTs. The specific peptides identified for CL1Contig7, CL1Contig2 and CL1Contig6 are indicated in red. (TIFF 2 MB)

Additional file 9: Figure S5: Partial multiple alignment of γ-GT sequences. The three *A. ervi* γ-GT sequences identified were aligned with related hymenopteran γ-GT sequences from the same clade (clade A in Additional file 4: Figure 4) and the human γ-GT1 sequence [Swiss-Prot:P19440]. The part of the multiple alignment displayed in the figure contains the mutations in the Aerv_CL1Contig6 that were described to affect the enzymatic activity of human γ-GT1. Mutations are indicated with stars and red letters. Residues identical or similar are highlighted in black and grey, respectively. Aech, *Acromyrmex echinatior*; Aerv, *Aphidius ervi*; Aflo, *Apis florea*; Amel, *Apis mellifera*; Bimp, *Bombus impatiens*; Bter, *Bombus terrestris*; Cflo, *Camponotus floridanus*; Hsal, *Harpegnathos saltator*; Hsap, *Homo sapiens*; Mrot, *Megachile rotundata*; Nvit, *Nasonia vitripennis.* (TIFF 5 MB)

Additional file 10: Figure S6: Multiple alignment of the five *A. ervi* serine protease homologue sequences. Residues identical or similar are highlighted in black and grey, respectively. Letters in red indicate residues of the catalytic triad (His, Asp and Ser) for which mutations are found in *A. ervi* serine protease homologue sequences*.* (TIFF 3 MB)

Additional file 11: Figure S7: Multiple alignment of LRR domain-containing sequences. Residues identical or similar are highlighted in black and grey, respectively. The predicted signal peptide is underlined in red. The 8 canonical LRR motifs are underlined in blue. (TIFF 2 MB)

Additional file 12: Figure S8: Multiple alignment of serpin sequences. The two *A. ervi* serpin sequences identified were aligned with *H. didymator* Hd-Ven390 [20] and *L. boulardi* LbSPNy [EMBL: ACQ83466.1] venom serpin sequences. Residues identical or similar are highlighted in black and grey, respectively. The hinge region is underlined in red. (TIFF 4 MB)

Additional file 13: Figure S9: Multiple alignment of endoplasmin sequences. The three *A. ervi* endoplasmin-like unisequences were aligned with *N. vitripennis* endoplasmin [GenBank: XP_001599282.1]. Residues identical or similar are highlighted in black and grey, respectively. (TIFF 2 MB)

Additional file 14: Table S5: Primer pairs used for qRT-PCR experiments. (DOC 32 KB)

## Abbreviations

EF-1α: Elongation factor 1-alpha
EF-2: Elongation factor 2
ER: Endoplasmic reticulum
EST: Expressed sequence tag
FR: French strain of A. ervi
γ-GT: γ -glutamyl transpeptidase
GO: Gene ontology
GSH: Glutathione
IT: Italian strain of A. ervi
LRR: Leucine-rich repeat
NEP: Neprilysin
NR: Non-redundant sequence database
Mr: Apparent molecular mass
PDV: Polydnavirus
RCL: Reactive center loop
SPH: Serine protease homolog.
