# Supplementary figures and images for: Identification of the main venom protein components of Aphidius ervi, a parasitoid wasp of the aphid model Acyrthosiphon pisum
Source: BMC Genomics. 2014 May 6;15(1):342. doi: 10.1186/1471-2164-15-342 (PMC4035087; doi:10.1186/1471-2164-15-342)

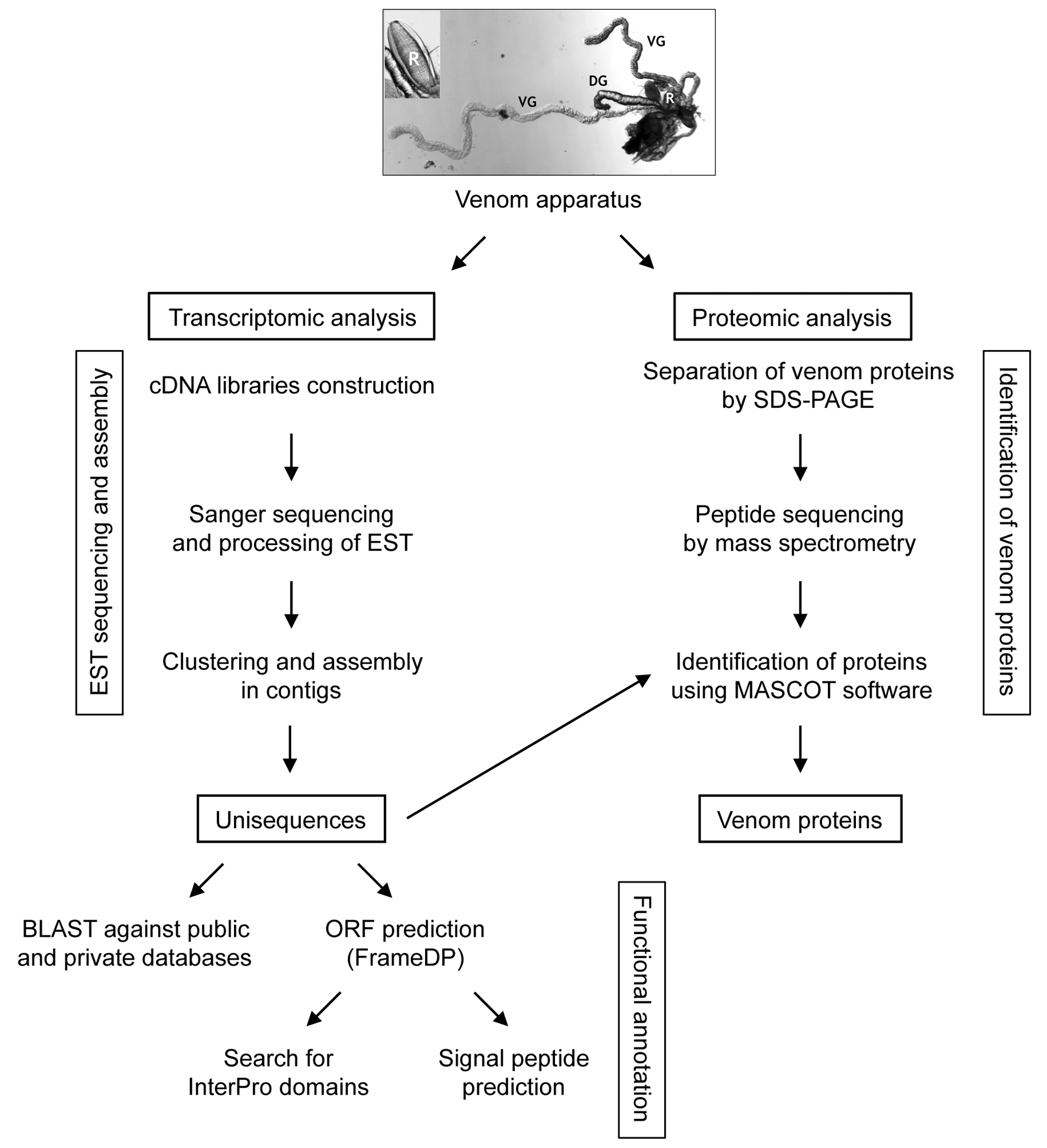

Supplement: Supplementary file 1 — Additional file 1: Figure S1: Schematic representation of the combined large-scale transcriptomic and proteomic approach. Upper picture; venom apparatus of A. ervi. VG: venom gland; R: reservoir; DG: Dufour gland. (TIFF 982 KB) [file 12864_2014_6064_MOESM1_ESM.tiff]

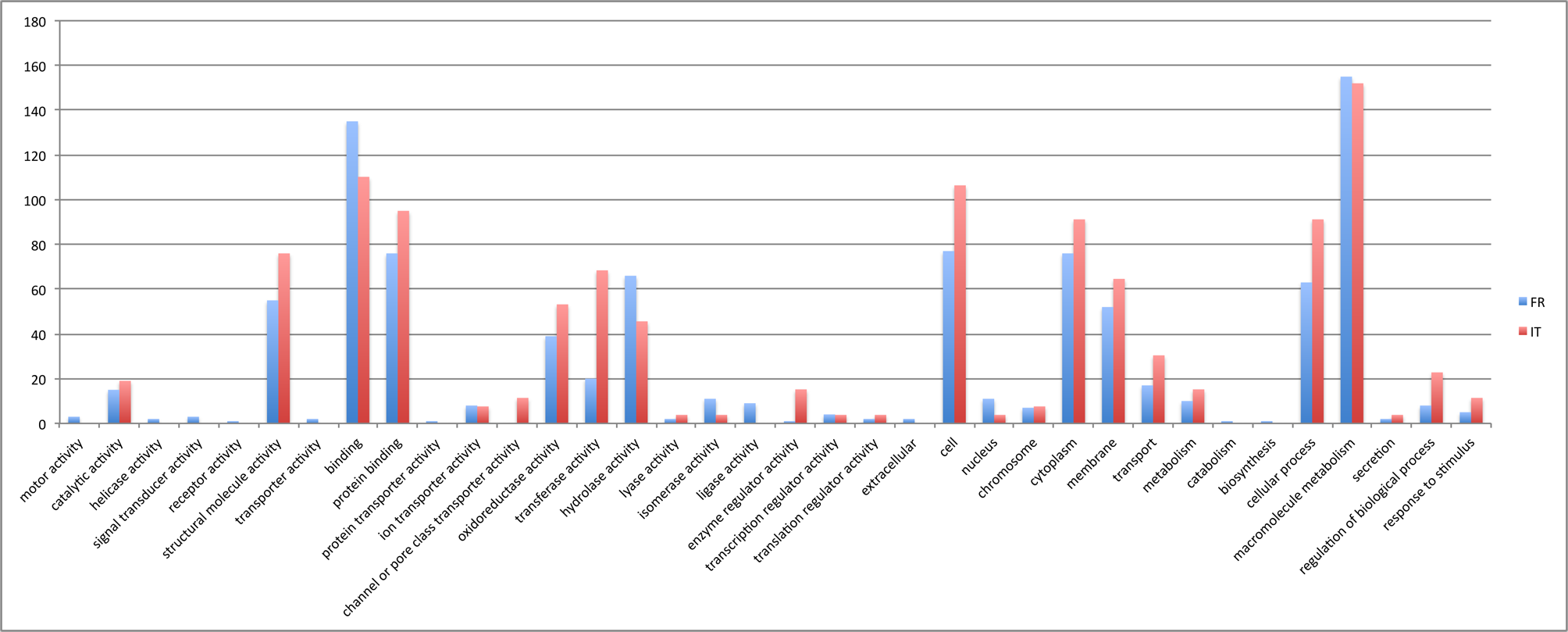

Supplement: Supplementary file 3 — Additional file 3: Figure S2: Interlibrary comparison of the representation of GO categories. Distribution of the number of unisequences associated with GO terms for the FR and IT libraries. The difference in the number of FR and IT sequences was taken into account using the ratio of the number of trimmed sequences between FR and IT. (TIFF 838 KB) [file 12864_2014_6064_MOESM3_ESM.tiff]

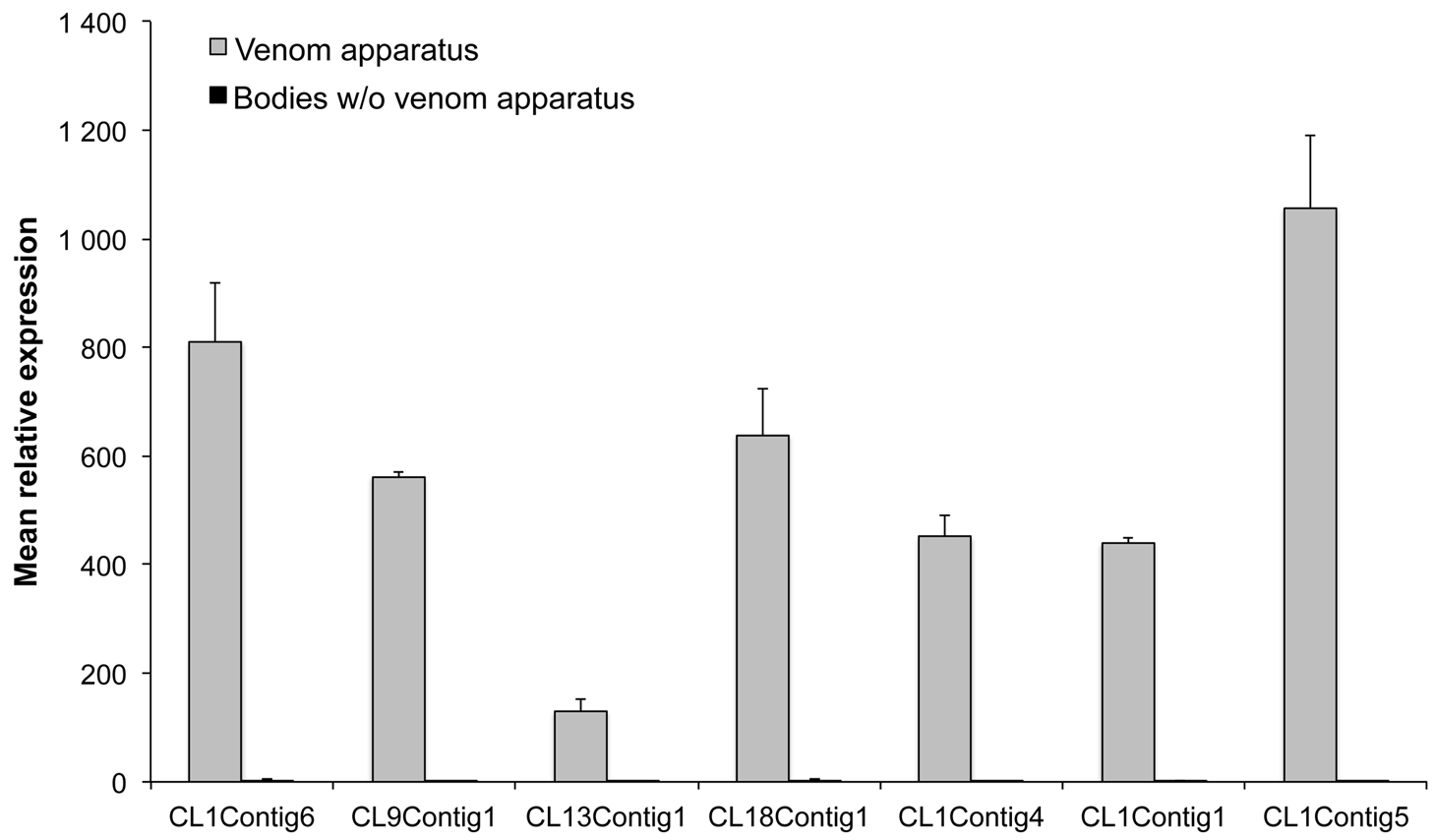

Supplement: Supplementary file 7 — Additional file 7: Figure S3: Mean relative expression in venom apparatus and bodies without venom apparatus. qRT-PCR experiments were performed for a selection of unisequences coding for putative venom proteins and toxin-like peptides. All data were normalized using RPL19 and RPL23 controls. (TIFF 526 KB) [file 12864_2014_6064_MOESM7_ESM.tiff]

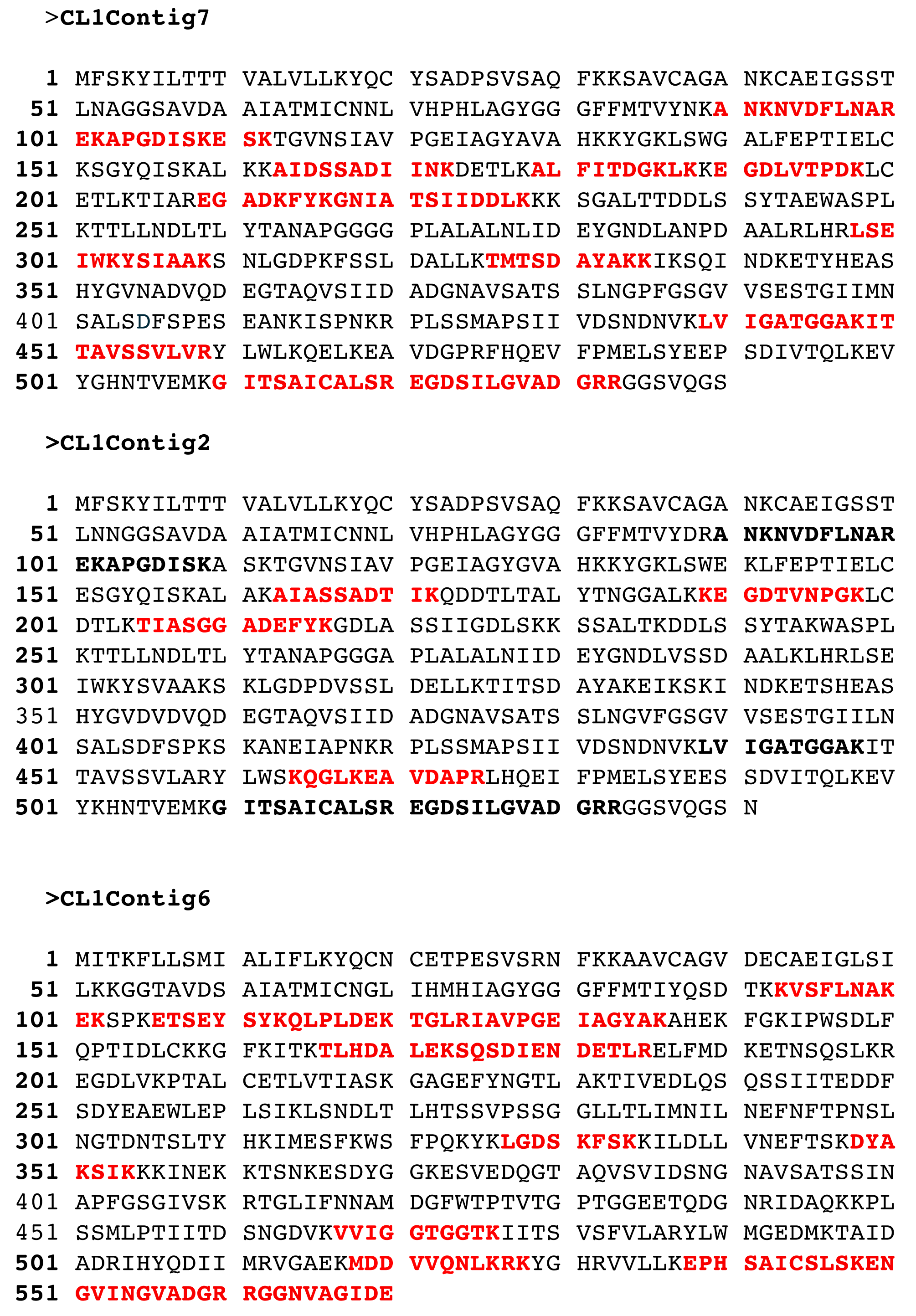

Supplement: Supplementary file 8 — Additional file 8: Figure S4: Specific peptides identified in proteomics for the three A. ervi venom γ-GTs. The specific peptides identified for CL1Contig7, CL1Contig2 and CL1Contig6 are indicated in red. (TIFF 2 MB) [file 12864_2014_6064_MOESM8_ESM.tiff]

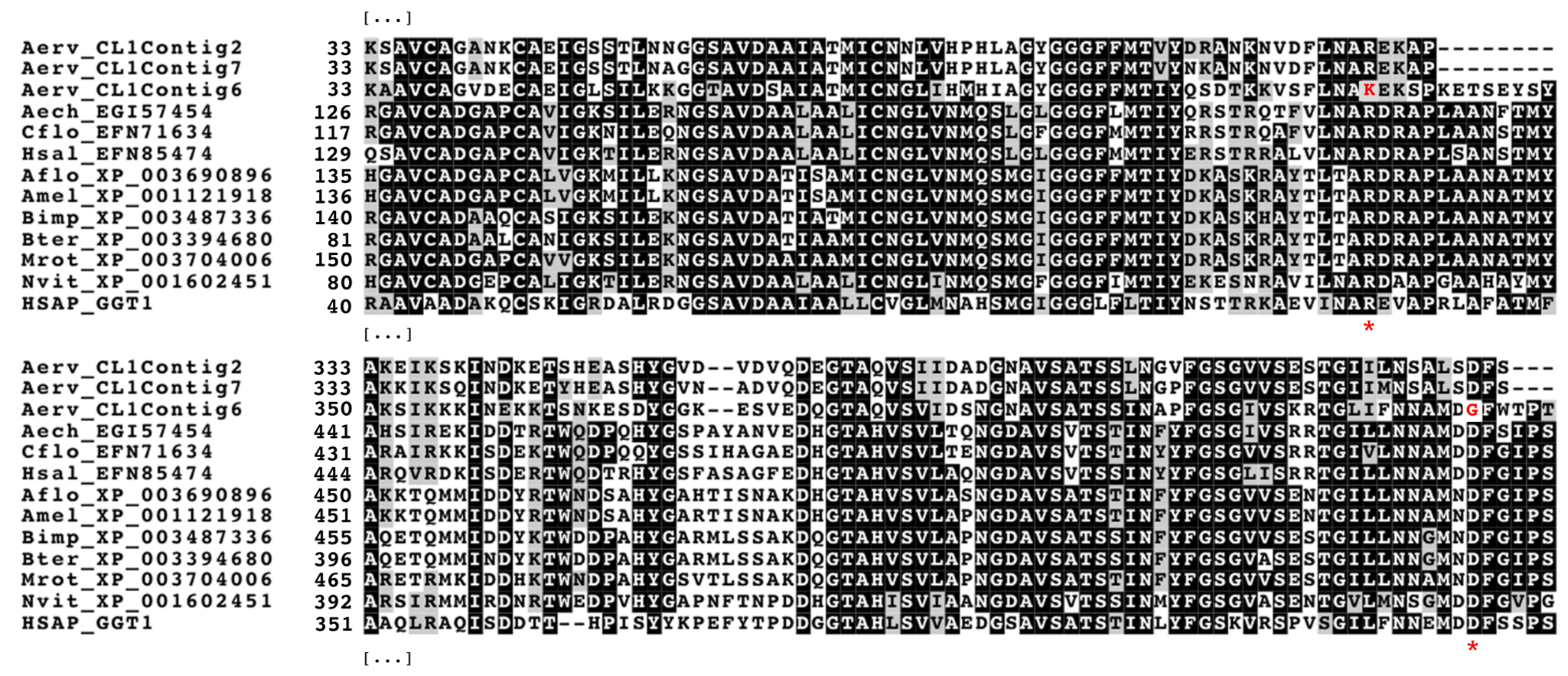

Supplement: Supplementary file 9 — Additional file 9: Figure S5: Partial multiple alignment of γ-GT sequences. The three A. ervi γ-GT sequences identified were aligned with related hymenopteran γ-GT sequences from the same clade (clade A in Additional file 4: Figure 4) and the human γ-GT1 sequence [Swiss-Prot:P19440]. The part of the multiple alignment displayed in the figure contains the mutations in the Aerv_CL1Contig6 that were described to affect the enzymatic activity of human γ-GT1. Mutations are indicated with stars and red letters. Residues identical or similar are highlighted in black and grey, respectively. Aech, Acromyrmex echinatior; Aerv, Aphidius ervi; Aflo, Apis florea; Amel, Apis mellifera; Bimp, Bombus impatiens; Bter, Bombus terrestris; Cflo, Camponotus floridanus; Hsal, Harpegnathos saltator; Hsap, Homo sapiens; Mrot, Megachile rotundata; Nvit, Nasonia vitripennis. (TIFF 5 MB) [file 12864_2014_6064_MOESM9_ESM.tiff]

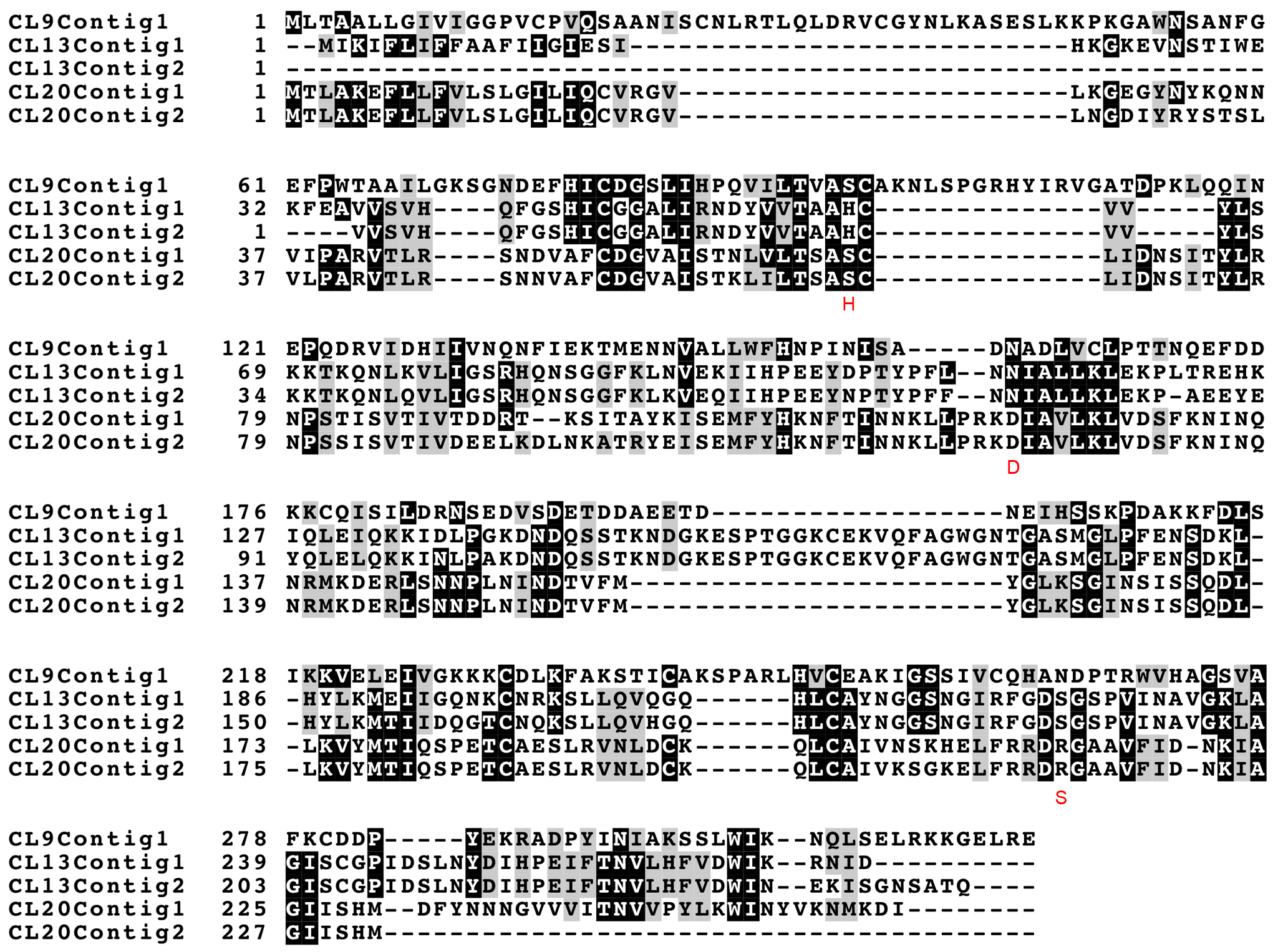

Supplement: Supplementary file 10 — Additional file 10: Figure S6: Multiple alignment of the five A. ervi serine protease homologue sequences. Residues identical or similar are highlighted in black and grey, respectively. Letters in red indicate residues of the catalytic triad (His, Asp and Ser) for which mutations are found in A. ervi serine protease homologue sequences. (TIFF 3 MB) [file 12864_2014_6064_MOESM10_ESM.tiff]

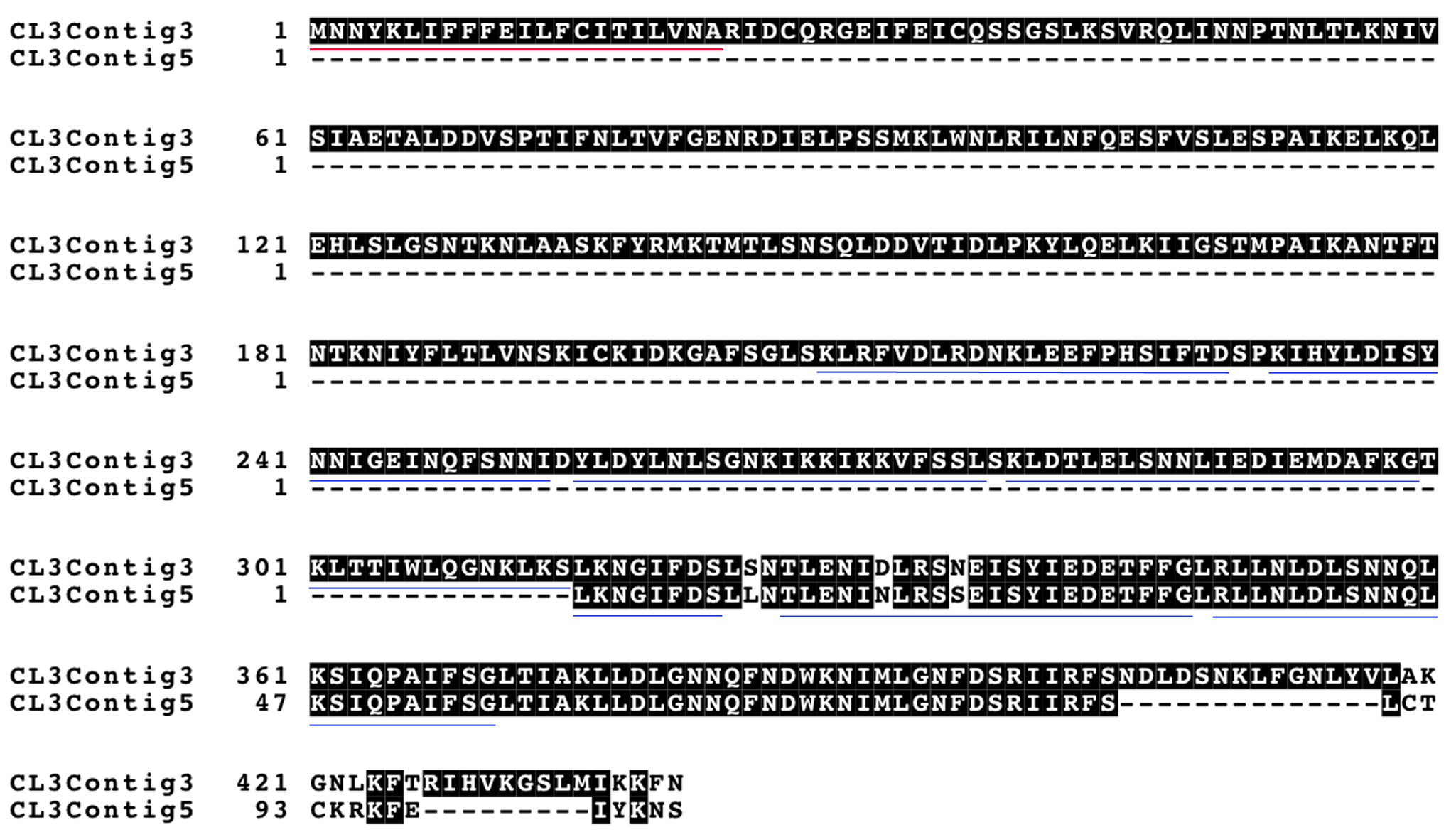

Supplement: Supplementary file 11 — Additional file 11: Figure S7: Multiple alignment of LRR domain-containing sequences. Residues identical or similar are highlighted in black and grey, respectively. The predicted signal peptide is underlined in red. The 8 canonical LRR motifs are underlined in blue. (TIFF 2 MB) [file 12864_2014_6064_MOESM11_ESM.tiff]

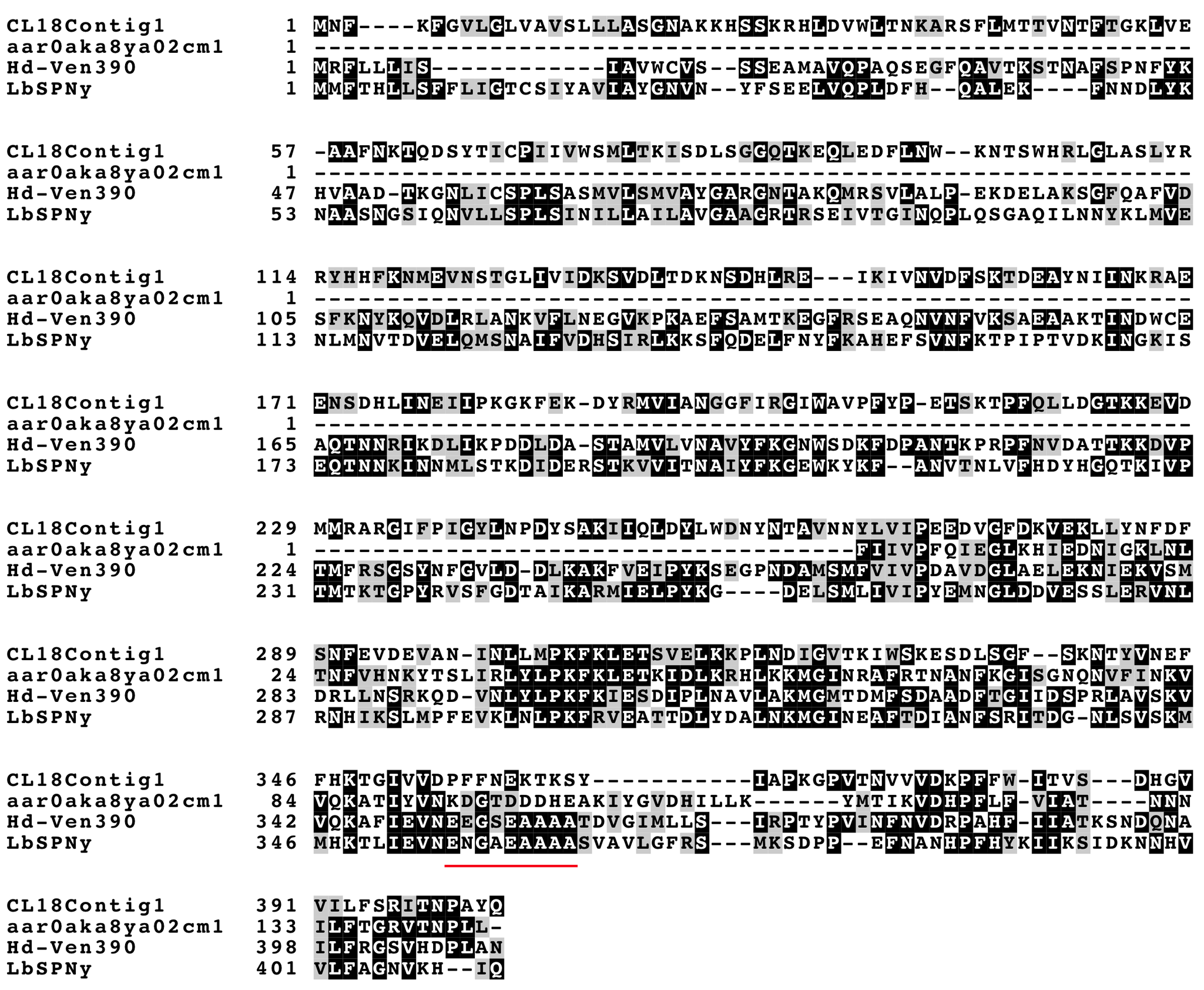

Supplement: Supplementary file 12 — Additional file 12: Figure S8: Multiple alignment of serpin sequences. The two A. ervi serpin sequences identified were aligned with H. didymator Hd-Ven390 [20] and L. boulardi LbSPNy [EMBL: ACQ83466.1] venom serpin sequences. Residues identical or similar are highlighted in black and grey, respectively. The hinge region is underlined in red. (TIFF 4 MB) [file 12864_2014_6064_MOESM12_ESM.tiff]

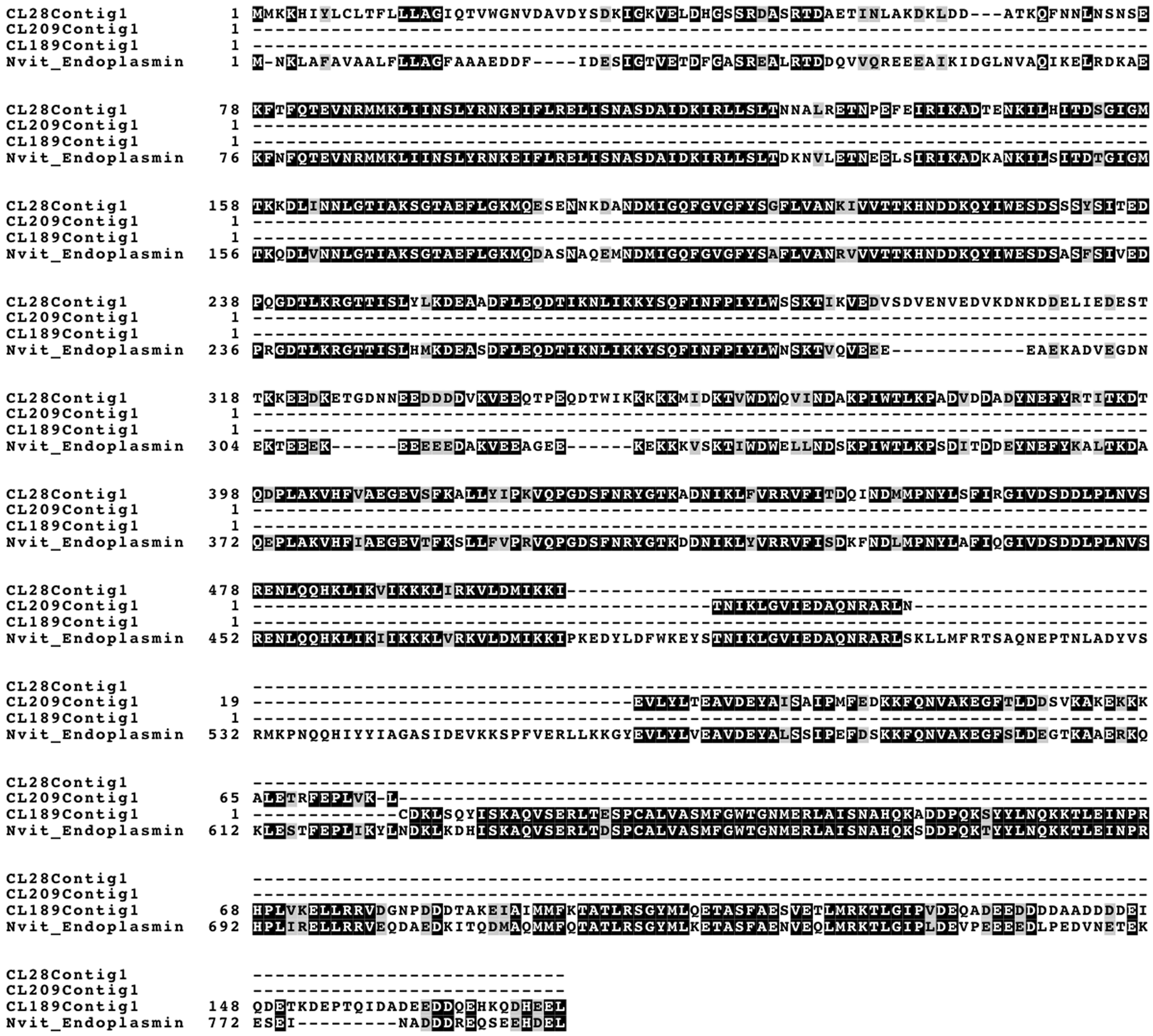

Supplement: Supplementary file 13 — Additional file 13: Figure S9: Multiple alignment of endoplasmin sequences. The three A. ervi endoplasmin-like unisequences were aligned with N. vitripennis endoplasmin [GenBank: XP_001599282.1]. Residues identical or similar are highlighted in black and grey, respectively. (TIFF 2 MB) [file 12864_2014_6064_MOESM13_ESM.tiff]
