# Supplementary material for: Identification of the main venom protein components of Aphidius ervi, a parasitoid wasp of the aphid model Acyrthosiphon pisum
Source: BMC Genomics. 2014 May 6;15(1):342. doi: 10.1186/1471-2164-15-342 (PMC4035087; doi:10.1186/1471-2164-15-342)
Supplement: Supplementary file 2 — Additional file 2: Table S1: General features of the A. ervi cDNA FR and IT libraries, results of assembly of pooled FR and IT sequences and similarity searches. (DOC 60 KB) [file 12864_2014_6064_MOESM2_ESM.doc]

**Table S1.** General features of the *A. ervi* cDNA FR and IT libraries, results of assembly of pooled FR and IT sequences and similarity searches.

|  | *A. ervi* FR | *A. ervi* IT | **Total** |
| --- | --- | --- | --- |
| DATA |  |  |  |
| Number of chromatograms | 4579 | 969 | **5548** |
| Number of trimmed sequences | 3499 | 928 | **4427** |

|  | Mixed contigs | FR only | IT only | **Total** |
| --- | --- | --- | --- | --- |
| ASSEMBLY OF POOLED FR AND IT SEQUENCES |  |  |  |  |
| Contigs (Number of ESTs) | 108 (1911) | 219 (906) | 13 (39) | **340 (2856)** |
| Singletons |  | 1220 | 351 | **1571** |
| Unisequences (% EST) | 108 (43.2%) | 1439 (48%) | 364 (8.8%) | **1911** |
| Average length (bp) |  |  |  | **510** |
| Max length (bp) |  |  |  | **2858** |
| Redundancy (%)^1^ |  |  |  | **56.8%** |
| REPARTITION OF CONTIGS |  |  |  |  |
| 2-10 ESTs | 70 | 210 | 12 | **292** |
| > 10 ESTs (% EST) | 38 (36.9%) | 9 (6.6%) | 1 (0.3%) | **48 (43.8%)** |

|  | **Total** |
| --- | --- |
| SIMILARITY SEARCHES |  |
| With public databases |  |
| NCBI NR | **770** |
| Swiss-Prot | **551** |
| With insect proteomes |  |
| *Acromyrmex echinatior* | **710** |
| *Acyrthosiphon pisum* | **580** |
| *Aedes aegypti* | **577** |
| *Anopheles gambiae* | **572** |
| *Apis mellifera* | **715** |
| *Bombyx mori* | **571** |
| *Drosophila melanogaster* | **582** |
| *Drosophila pseudoobscura* | **567** |
| *Nasonia vitripennis* | **699** |
| *Tribolium castaneum* | **615** |

| TRANSLATION AND SECRETION^2^ |  |
| --- | --- |
| Unisequences with ORF prediction | **1008** |
| Unisequences with signal peptide | **132** |
| SEARCH FOR INTERPRO DOMAINS |  |
| Unisequences with IPR | **675** |
| Unisequences with GO | **510** |

^1^ Redundancy was estimated by: 1 - number of unisequences/number of trimmed sequences

^2^ ORF prediction was obtained using FrameDP (Gouzy et al 2009). Signal peptide prediction was obtained using SignalP software available at http://www.cbs.dtu.dk/services/SignalP/.
