# Supplementary material for: Identification of the main venom protein components of Aphidius ervi, a parasitoid wasp of the aphid model Acyrthosiphon pisum
Source: BMC Genomics. 2014 May 6;15(1):342. doi: 10.1186/1471-2164-15-342 (PMC4035087; doi:10.1186/1471-2164-15-342)
Supplement: Supplementary file 14 — Additional file 14: Table S5: Primer pairs used for qRT-PCR experiments. (DOC 32 KB) [file 12864_2014_6064_MOESM14_ESM.doc]

**Table S5.** Primer pairs used for qRT-PCR experiments.

| Unisequence name | Forward primer | Reverse primer |
| --- | --- | --- |
| CL1Contig6 | TAACGCTCCCTTTGGAAGTG | TGAGTTTCTTCGCCTCCAGT |
| CL9Contig1 | CCAACTCGTTGGGTACATGC | CGCAATTCGCTTAATTGGTT |
| CL13Contig1 | TGGCGGATCAAATGGTATTC | TCCACAGCTAATGCCAGCTA |
| CL18Contig1 | TGGGATTGGTTGCTGTAAGTC | TCCACAAGCTTGCCAGTAAA |
| CL1Contig4 | GGCTGCAGGAGACTTGGTAG | CCATTATCTCCATCAATTGGTTT |
| CL1Contig1 | TGGAGCTAAAGCTGATGCAGT | CGATGCAGCATTTGTTGAGT |
| CL1Contig5 | TGCATACTGCCTAGGATCTGG | TGCACATTCTCCATTTGACA |
| RPL19 | ATCAAGCTGAAGCTCGTCGT | TGCAGCTGCTTCATCTTCAC |
| RPL23 | CGCTGGAGCTAAATTCAGGA | GCAGCTGGGAGACGATTAAG |
